# Early-life Exposure to Organophosphate Pesticides and Pediatric Respiratory Symptoms in the CHAMACOS Cohort

**DOI:** 10.1289/ehp.1408235

**Published:** 2014-11-04

**Authors:** Rachel Raanan, Kim G. Harley, John R. Balmes, Asa Bradman, Michael Lipsett, Brenda Eskenazi

**Affiliations:** 1 Center for Environmental Research and Children’s Health (CERCH), School of Public Health, University of California, Berkeley, California, USA; 2 Division of Environmental Health Sciences, School of Public Health, University of California, Berkeley, Berkeley, California, USA; 3 Divison of Occupational and Environmental Medicine, University of California, San Francisco, San Francisco, California, USA; 4 California Department of Public Health, Richmond, California, USA

## Abstract

Background: Although pesticide use is widespread, the possible effect of early-life exposure to organophosphate (OP) on pediatric respiratory health is not well described.

Objectives: We investigated the relationship between early-life exposure to OPs and respiratory outcomes.

Methods: Participants included 359 mothers and children from the CHAMACOS birth cohort. Dialkyl phosphate (DAP) metabolites of OP pesticides, specifically diethyl (DE) and dimethyl (DM) phosphate metabolites, were measured in urine from mothers twice during pregnancy (mean = 13 and 26 weeks gestation) and from children five times during childhood (0.5–5 years). Childhood DAP concentrations were estimated by the area under curve (AUC). Mothers reported their child’s respiratory symptoms at 5 and 7 years of age. We used generalized estimating equations (GEE) to examine associations of prenatal and childhood DAP concentrations with repeated measures of respiratory symptoms and exercise-induced coughing at 5 and 7 years of age, adjusting for child’s sex and age, maternal smoking during pregnancy, secondhand tobacco smoke, season of birth, PM_2.5_, breastfeeding, mold and cockroaches in home, and distance from highway.

Results: Higher prenatal DAP concentrations, particularly DE, were nonsignificantly associated with respiratory symptoms in the previous 12 months at 5 or 7 years of age [adjusted odds ratio (aOR) per 10-fold increase = 1.44; 95% CI: 0.98, 2.12]. This association was strongest with total DAP and DE from the second half of pregnancy (aOR per 10-fold increase = 1.77; 95% CI: 1.06, 2.95; and 1.61; 95% CI: 1.08, 2.39, respectively). Childhood DAP, DE, and DM concentrations were associated with respiratory symptoms and exercise-induced coughing in the previous 12 months at 5 or 7 years of age (total DAPs: aOR per 10-fold increase = 2.53; 95% CI: 1.32, 4.86; and aOR = 5.40; 95% CI: 2.10, 13.91, respectively).

Conclusions: Early-life exposure to OP pesticides was associated with respiratory symptoms consistent with possible asthma in childhood.

Citation: Raanan R, Harley KG, Balmes JR, Bradman A, Lipsett M, Eskenazi B. 2015. Early-life exposure to organophosphate pesticides and pediatric respiratory symptoms in the CHAMACOS cohort. Environ Health Perspect 123:179–185; http://dx.doi.org/10.1289/ehp.1408235

## Introduction

Asthma is the most prevalent pediatric chronic disease [Pijnenburg 2012; World Health Organization (WHO) 2007], and is a leading cause of hospitalization in children (Mellon and Parasuraman 2004) and school absenteeism due to chronic disease (Mellon and Parasuraman 2004). It is estimated that by 2025, > 350 million people globally, mostly children, will have asthma (Pawankar et al. 2011; WHO 2007). Early-life exposures to maternal smoking, secondhand tobacco smoke, and various ambient air pollutants have been linked to respiratory symptoms and disease in childhood (Pawankar et al. 2011; Selgrade et al. 2013; WHO 2007) and adulthood (Stocks and Sonnappa 2013; WHO 2007). The impact of early-life exposures on later respiratory health is biologically plausible: During the first half of gestation, bronchi are developing and airways are branching; during the second half of gestation, alveoli begin to develop; and for several years after birth, the lungs continue to mature with rapid increase in number, size, and complexity of the alveoli (De Luca et al. 2010).

Organophosphate pesticides (OPs) are one of the most commonly used classes of insecticides worldwide. The U.S. Environmental Protection Agency (EPA) phased out most residential use of OP pesticides by the mid-2000s. However, in 2007, 15 million kg of OPs—36% of total insecticide use—were applied in agriculture in the United States (Grube et al. 2011; Guha et al. 2013; U.S. EPA 2013). Widespread OP exposure in the general U.S. population is supported by the frequent detection of diakyl phosphates (DAPs), urinary metabolites of OP pesticides, in the U.S. National Health and Nutrition Examination Survey (NHANES) (Bradman et al. 2005; CDC 2004, 2014).

OPs depress acetylcholinesterase (AChE), allowing acetylcholine to build up in neuronal junctions, including those of the parasympathetic nervous system, which helps modulate control of the airways (Barnes 1986). In animal studies, the OPs—chlorpyrifos, parathion, and diazinon—induced airway hyperreactivity at doses below those causing AChE inhibition (Fryer et al. 2004; Lein and Fryer 2005; Ndlovu et al. 2011; Proskocil et al. 2013). OP exposure has been associated with respiratory symptoms in adults in occupational settings (Hoppin et al. 2006; Kwak et al. 2009; Ndlovu et al. 2011) and in case studies of children following pesticide poisonings (Cavari et al. 2013); however, there have been few investigations of respiratory symptoms following low-level exposure. One nested case–control study (Salam et al. 2004) reported an association between maternal report of exposure to pesticides and herbicides in the first year of life and asthma before 5 years of age. A cross-sectional study of Lebanese children 5–16 years of age (Salameh et al. 2003) reported an association between parental report of para-occupational and residential exposure to pesticides and respiratory symptoms. As noted by others (Kwak et al. 2009; Ndlovu et al. 2011), these studies were based on reported exposure to pesticides, and no studies of children’s respiratory health have included biological measures of exposure.

Here we investigate associations between maternally reported respiratory symptoms consistent with possible asthma and pre- and postnatal exposure to OPs, as measured by DAP metabolite concentrations in urine samples collected from pregnant women and their children from an agricultural community in California. We previously reported an association between maternal work in agriculture and increased levels of Th2 (T helper cell) cytokines in these children at age 2 years, which likely play a key role in the pathophysiology of allergic diseases, including childhood asthma (Duramad et al. 2006).

## Methods

*Study setting and design*. The Center for the Health Assessment of Mothers and Children of Salinas (CHAMACOS) study is a longitudinal birth cohort investigating the effects of *in utero* and postnatal environmental exposures on growth, neurodevelopment, and respiratory disease in residents of the Salinas Valley, California (Eskenazi et al. 2007). Approximately 235,000 kg of OP pesticides were applied in this agricultural valley during the years of enrollment [California Department of Pesticide Regulation (CDPR) 2001]. Detailed methods have been described elsewhere (Eskenazi et al. 2007). In brief, pregnant women were screened for eligibility between October 1999 and 2000 at community clinics primarily serving farmworker families. Women were eligible for enrollment if they were ≥ 18 years old, < 20 weeks gestation, Spanish- or English-speaking, eligible for low-income health insurance, receiving prenatal care, and planning to deliver at the local county hospital. Research protocols were approved by the University of California, Berkeley, Committee for the Protection of Human Subjects. Written informed consent was obtained from the mothers and verbal assent was obtained from the children at age 7 years.

A total of 601 pregnant women were enrolled in the CHAMACOS cohort. Of these, 526 delivered live-born surviving singletons. We assessed respiratory symptomatology for 344 of these children at 5 years of age and 347 at 7 years, with 327 assessed at both time points and 364 children assessed at least once. A total of 359 mothers of the 364 children had a urinary DAP measurement during their pregnancy.

*Maternal interviews and respiratory symptom assessment*. Information on respiratory symptoms and relevant covariates was obtained by maternal interviews and home visits. Mothers were interviewed twice during pregnancy (mean ± SD = 13.5 ± 4.8 and 26.4 ± 2.4 weeks gestation), after delivery, and when children were 0.5, 1, 2, 3.5, 5, and 7 years old. Urine samples were collected at each prenatal visit and at each child visit, except at 7 years, and stored at –80°C. Homes were inspected by trained personnel when the children were 6 and 12 months old. Additional data from prenatal and delivery records were abstracted by a registered nurse.

For the present study, we used maternal report of the child’s respiratory symptoms when the child was 5 and 7 years of age. Mothers were asked questions based on the International Study of Asthma and Allergies in Childhood (ISAAC) questionnaire (Asher et al. 1995; Holguin et al. 2007; Kraai et al. 2013; Stellman et al. 2013). Additionally, mothers were asked whether the child had been prescribed any medication for asthma or wheezing/whistling, or tightness in the chest. We defined respiratory symptoms as a binary outcome based on a positive response to any of the following during the previous 12 months: *a*) wheezing or whistling in the chest; *b*) wheezing, whistling, or shortness of breath so severe that the child could not finish saying a sentence; *c*) trouble going to sleep or being awakened from sleep because of wheezing, whistling, shortness of breath, or coughing that was not associated with a cold; or *d*) having to stop running or playing active games because of wheezing, whistling, shortness of breath, or coughing that was not associated with a cold. In addition, a child was included as having respiratory symptoms if the mother reported use of asthma controller or rescue medications, even in the absence of the above symptoms. We also analyzed separately the binary outcome of maternal report of the child having to stop running or playing active games due to coughing that was not associated with a cold in the previous 12 months (i.e., exercise-induced coughing). Exercise-induced coughing was included under the respiratory symptoms variable—all children with exercise-induced coughing were also classified as having respiratory symptoms. Children who were not categorized as positive for exercise-induced coughing but had other respiratory symptoms were classified as noncases for these analyses and were not excluded from the analyses. Exercise-induced coughing was analyzed separately without including coughing that was associated with sleep (either trouble going to sleep or being awakened from sleep) because coughing associated with sleep issues may be related to health conditions other than asthma. We did not analyze wheezing separately because of the relatively small percentage of mothers who reported on wheezing.

*OP pesticide exposure: DAP metabolites*. Six nonspecific DAP metabolites—three dimethyl phosphate (DM) and three diethyl phosphate (DE) metabolites—were measured in urine samples collected from mothers twice during pregnancy and from children at 0.5, 1, 2, 3.5, and 5 years of age. Analyses were conducted by the Division of Laboratory Science at the Centers for Disease Control and Prevention (CDC) using gas chromatography–tandem mass spectrometry and quantified using isotope dilution calibration (Bravo et al. 2002). Detailed methods of urine sample collection and analysis are described elsewhere (Bradman et al. 2005). To account for urine dilution, we measured specific gravity using a hand-held refractometer (National Instrument Company Inc., Baltimore, MD) and measured creatinine concentration using a commercially available diagnostic assay (Vitros CREA slides; Ortho Clinical Diagnostics, Raritan, NJ). The individual DAP metabolites were summed on a molar basis to yield total DAPs, as well as total DE and DM metabolites (Bradman et al. 2005). These metabolites are biomarkers for about 80% of OP pesticides used in the Salinas Valley (CDC 2009). In 2001, the most commonly used OPs in the Salinas Valley that metabolized to DEs were diazinon (60,571 kg) and chlorpyrifos (24,923 kg) and to DMs were malathion (43,781 kg) and oxydemeton methyl (26,244 kg) (CDPR 2001).

*Data analysis*. We examined the relationship of DEs, DMs, and total DAP concentrations (nanomoles per liter) from maternal urine collected during the first and second halves of pregnancy (0–20, 21–40 weeks gestation), and the average of the two pregnancy samples. For childhood metabolite concentrations, we used the area under the curve (AUC) from the five measurements made during childhood to summarize DAP concentrations over time during childhood. We calculated the time-weighted average concentration for each time interval by multiplying the time between measurements in years by the average of the two measured concentrations. The AUC was calculated by summing the time-weighted averages from each time interval using the trapezoidal method. We excluded from the AUC calculation 86 children who were missing DAP measurements at either 6 months or 5 years or missing more than one measurement from the other three time points. For children with a single missing DAP measurement at 1, 2, or 3.5 years of age (*n* = 65), we derived the time-weighted average for the interval defined by time points with available data by calculating the mean of the two closest measures. A total of 270 children had a childhood AUC calculation and data on the relevant covariates. We also performed a sensitivity analysis of the association between respiratory symptoms during the previous year reported at 5 or 7 years of age and a summary of childhood OP exposures based on measured values through 3.5 years of age only. In this analysis we excluded the concurrent 5-year value to ensure that the DAP measurements preceded the respiratory symptoms and exercise-induced coughing outcomes. DAP concentrations in maternal samples were corrected for urinary dilution using urine specific gravity, and child DAP samples were corrected for urinary dilution by dividing by urinary creatinine concentration. The variables for total concentrations of DEs, DMs, and total DAPs were log_10_-transformed.

We used generalized estimating equation (GEE) models (Hubbard et al. 2010; Zeger and Liang 1986) to estimate the longitudinal associations of prenatal and early childhood DAPs on respiratory symptoms in children at 5 and 7 years of age, while accounting for within-subject correlation of repeated measures of respiratory symptoms that were assessed at 5 and 7 years of age (Hubbard et al. 2010; Zeger and Liang 1986). The repeated measures of respiratory symptoms were assessed at 5 and 7 years of age and were defined as positive if they were categorized as such at age 5 or 7 years or both. The same definition was done for exercise-induced coughing.

Covariates were selected based on directed acyclic graphs (DAGs) and included in models if associated with respiratory symptoms in bivariate analysis (*p* < 0.25). Final adjusted models controlled for child’s sex, maternal smoking during pregnancy (yes/no), exposure to secondhand tobacco smoke in the first year of life (yes/no), season of birth (wet/pollen/dry/mold), mean daily particulate matter concentrations with aerodynamic diameter ≤ 2.5 μm (PM_2.5_) during first 3 months of life, breastfeeding duration (months), signs of moderate or extensive mold noted at either home visit (6 and 12 months), home located ≤ 150 m from a highway in first year of life (based on geographic information system), and signs of cockroaches noted at home visit (6 and 12 months). We also controlled for the child’s age in months. Season of birth corresponds generally, but not exactly, to mold = fall, wet = winter, pollen = spring, dry = summer. Discrete seasons of high spore and pollen concentrations were determined by ambient aeroallergen concentrations that were measured throughout the birth periods of the participants. Detailed methods for the differentiation of the four seasons have been described elsewhere (Harley et al. 2009). Average PM_2.5_ concentration in the first 3 months of life was calculated using data from the Monterey Unified Air Pollution Control District (MBAPCD) air monitoring station, which uses high-volume Sierra-Andersen gravimetric samplers for 24 hr every sixth day (Thermo Scientific, Waltham, MA). We conducted sensitivity analyses to verify the robustness and consistency of our findings. Models were re-run without adjusting for specific gravity (maternal DAPs) or urine creatinine (child DAPs). Additional models were run controlling for both prenatal DAP levels (we analyzed average and first and second halves of pregnancy measurements separately) and DAP concentrations measured during childhood (calculated by the AUC) in the same model. Potential selection bias due to exclusion from final models of children with missing outcome data or missing covariates was addressed by comparing our results to GEE models that included stabilized inverse probability weights (Hernán et al. 2004). Weights were determined using multiple logistic regression with independent demographic variables selected based on a “Super Learner” algorithm using V-fold cross-validation (van der Laan et al. 2007). Estimates for both weighted and unweighted regression models yielded similar results (data not shown), suggesting that selection bias did not substantially modify our results.

We analyzed the data using SPSS (version 20.0; IBM Corp., Somers, NY) for bivariate analyses, Stata (version IC11.2; StataCorp, College Station, TX) for GEE models, and R (v.2.14.2; R Foundation for Statistical Computing, Vienna, Austria) for Super Learner models. We set statistical significance at *p* < 0.05 for all analyses.

## Results

Characteristics of the CHAMACOS cohort are shown in Table 1. The cohort subjects were primarily born of mothers who were from Mexico, had less than a high school education, and lived in families with income at or below the federal poverty level. A total of 78% of pregnant mothers and about 70% of 5- and 7-year-olds lived in a household with at least one farm worker. The geometric mean (GM) of total DAP, DE, and DM specific gravity–adjusted concentrations during pregnancy were 147, 24, and 106 nmol/L, respectively (Table 2). Child creatinine-adjusted total DAP concentrations decreased with age, averaging 205, 233, 216, 152, and 131 nmol/g-creatinine at 0.5, 1, 2, 3.5, and 5 years of age, respectively (Table 2). The geometric mean (GM) of total DAP, DE, and DM creatinine-adjusted concentrations during childhood as measured by the AUC were 1,655, 259, and 1,281 nmol/year/g-creatinine (Table 2). Maternal DAP metabolites were not correlated with childhood measurements (total DAPs: *r* = –0.01 to 0.01, *p* = 0.84 to 0.95; DEs: *r* = –0.07 to –0.01, *p* = 0.25 to 0.93; DMs: *r* = 0.00 to 0.02, *p* = 0.7 to 0.97). A total of 25.9% and 16.1% of 5- and 7-year-olds, respectively, were reported to have respiratory symptoms or to be taking controller or rescue medication during the previous 12 months (see Supplemental Material, Table S1). A total of 11.1% and 3.8% of 5- and 7-year-olds, respectively, were reported to have had to stop running or playing active games because of coughing that was not associated with a cold during the previous 12 months (i.e., exercise-induced coughing) (see Supplemental Material, Table S1).

**Table 1:** Sociodemographic and household characteristics, CHAMACOS cohort, California (*n* = 364).

Characteristic	*n* (%)
Child’s sex
Boys	174 (47.8)
Girls	190 (52.2)
Season of birth
Mold	131 (36.0)
Wet	68 (18.7)
Pollen	80 (22.0)
Dry	85 (23.3)
Breastfeeding duration
Never breastfed	17 (4.7)
≤ 6 months	174 (47.8)
> 6 months	173 (47.5)
Mother’s country of birth
Mexico	314 (86.2)
United States	45 (12.4)
Other	5 (1.4)
Maternal education
≤ 6th grade	166 (45.6)
7–12th grade	125 (34.3)
Completed high school	73 (20.1)
Maternal history of asthma
Yes	17 (4.7)
No	346 (95.3)
Mother smoked during pregnancy
Yes	15 (4.1)
No	349 (95.9)
Agricultural workers in the household during pregnancy
Yes	283 (77.8)
No	81 (22.3)
Family income at 5 and 7 years
< Poverty level	294 (80.8)
≥ Poverty level	70 (19.2)
Infant around smokers (0–12 months)
Yes	29 (8.1)
No	329 (91.9)
Home ≤ 150 m from highway (6 or 12 months)
Yes	17 (5.1)
No	319 (94.9)
Mean daily PM_2.5_ near home (0–3 months)
< 8 μg/m^3^	175 (48.1)
8–12 μg/m^3^	149 (40.9)
≥ 12 μg/m^3^	40 (11.0)
Signs of rodents at home visit (6 or 12 months)
Yes	141 (42.0)
No	195 (58.0)
Signs of cockroaches at home visit (6 or 12 months)
Yes	224 (66.7)
No	112 (33.3)
Signs of moderate/extensive mold at home visit (6 or 12 months)
Yes	220 (65.5)
No	116 (34.5)
Gas stove in home (6 or 12 months)
Yes	298 (83.2)
No	60 (16.8)
Agricultural workers in the household at 5 years
Yes	241 (70.1)
No	103 (29.9)
Agricultural workers in the household at 7 years
Yes	235 (67.7)
No	112 (32.3)
A total of 344 and 347 children were assessed at 5 and 7 years, respectively. A total of 327 children were assessed for respiratory symptoms at both time points (at 5 and 7 years of age), and 364 children were assessed at least once. Information was missing for the following covariates: maternal history of asthma (*n *= 1), infant around smokers (*n *= 6), distance of home from highway (*n *= 28), signs of rodents, cockroaches, and mold (6 or 12 months; *n *= 28), and gas stove at home (*n *= 6).	

**Table 2:** DAP metabolite concentrations, measured in maternal urine during pregnancy (nmol/L) and in children’s urine at follow-up visits (nmol/g-creatinine) between 0.5 and 5 years of age, CHAMACOS.

Measurements of pregnancy (nmol/L)	*n*	DF (%)	GM (95% CI)	Minimum	25th	50th	75th	90th	Maximum
First half of pregnancy
Total DAPs	262	90.1	111 (93, 133)	4	40	107	312	826	5,026
DE	262	76.7	16 (13, 19)	0.2	6	13	39	95	2,436
DM	262	84.0	74 (61, 90)	2	21	75	234	622	5,019
Second half of pregnancy
Total DAPs	338	99.4	126 (113, 141)	6	68	123	235	499	2,366
DE	338	98.2	21 (18, 24)	0.7	8	22	51	124	630
DM	339	99.4	86 (76, 97)	2	40	88	175	388	3,175
Pregnancy average
Total DAPs	359	100	147 (132, 163)	10	75	141	301	577	2,555
DE	359	100	24 (21, 27)	0.4	11	25	51	99	1,245
DM	360	100	106 (94, 119)	5	51	97	231	504	3,175
At 6 months
Total DAPs	320	99.1	205 (172, 243)	2	76	184	608	1,702	78,235
DE	320	89.7	38 (31, 47)	0.1	13	56	132	292	78,010
DM	320	88.8	97 (79, 119)	0.7	29	84	338	1,511	10,073
At 1 year
Total DAPs	331	95.8	233 (197, 275)	4	80	222	655	1,752	10,552
DE	331	93.1	61 (53, 70)	0.8	31	69	140	254	1,972
DM	331	79.5	112 (91, 139)	0.8	28	118	484	1,223	10,298
At 2 years
Total DAPs	325	96.3	216 (183, 254)	3	93	223	592	1,389	5,943
DE	325	71.4	23 (18, 30)	0.0	3	47	120	324	3,926
DM	325	96.0	148 (125, 176)	2	53	158	435	1,009	5,843
At 3.5 years
Total DAPs	262	94.3	152 (126, 184)	2	53	174	448	933	9,240
DE	262	60.3	5 (4, 7)	0.0	0.6	11	52	146	546
DM	262	92.8	125 (103, 152)	2	43	141	348	867	8,694
At 5 years
Total DAPs	313	91.4	131 (110, 156)	0.9	47	148	346	840	10,085
DE	313	52.4	3 (2, 4)	0.0	0.3	6	42	113	634
DM	313	88.5	102 (85, 123)	0.8	40	113	288	772	10,052
AUC (0.5–5 years; nmol/year/g-crt)
Total DAPs	278	91.0–98.9	1,655 (1,482, 1,849)	118	836	1,636	3,048	6,335	18,927
DE	278	52.5–92.3	259 (229, 292)	14	136	251	529	966	16,580
DM	278	78.0–96.7	1,281 (1,139, 1,440)	79	605	1,244	2,510	5,636	15,460
Abbreviations: crt, creatinine; DF, detection frequency. 25th, 50th, 75th, and 90th are percentiles. Limits of detection for all DE analytes ranged from 0.05 to 0.2 ug/L, and for all DM analytes, 0.08 to 0.58 ug/L. Pregnancy measurements were specific gravity adjusted, and childhood measurements were creatinine adjusted.									

Total average DAPs and DM urinary concentrations during pregnancy were not significantly associated with reported respiratory symptoms assessed at 5 and 7 years (Table 3). However, higher prenatal DE concentrations were nonsignificantly associated with increased odds of respiratory symptoms [adjusted odds ratio (aOR) for a 10-fold increase in concentration = 1.44; 95% confidence interval (CI): 0.98, 2.12, *p* = 0.07]. Prenatal total DAPs, DE, and DM concentrations were not significantly associated with exercise-induced coughing. Examining timing of exposure, we found no associations with DAP concentrations in the first half of pregnancy, but significantly increased odds of respiratory symptoms in the children with total DAPs and DE metabolites from the second half of pregnancy (aOR for a 10-fold increase in concentration = 1.77; 95% CI: 1.06, 2.95, *p* = 0.03; aOR = 1.61; 95% CI: 1.08, 2.39, *p* = 0.02, respectively) (Table 3). DM metabolites from the second half of pregnancy were not significantly associated with respiratory symptoms.

**Table 3:** Associations [aOR (95% CI)]^*a,b *^of repeated measures of respiratory outcomes at 5 and 7 years of age with maternal urinary DAP metabolites (nmol/L) collected at pregnancy.*^c,d^*

Timing of measurement	*n*	Respiratory symptoms	*p*-Value	Exercise-induced coughing^*e*^	*p*-Value
First half of pregnancy
Total DAPs	241	1.11 (0.72, 1.72)	0.63	1.24 (0.58, 2.66)	0.59
DEs	241	1.03 (0.64, 1.65)	0.91	0.86 (0.46, 1.60)	0.64
DMs	241	1.08 (0.74, 1.58)	0.69	1.19 (0.60, 2.37)	0.62
Second half of pregnancy
Total DAPs	313	1.77 (1.06, 2.95)	0.03	1.25 (0.50, 3.11)	0.64
DEs	313	1.61 (1.08, 2.39)	0.02	1.20 (0.70, 2.04)	0.50
DMs	313	1.45 (0.90, 2.33)	0.12	1.28 (0.56, 2.94)	0.56
Pregnancy average^*d*^
Total DAPs	331	1.28 (0.77, 2.13)	0.34	1.14 (0.47, 2.74)	0.77
DEs	331	1.44 (0.98, 2.12)	0.07	0.94 (0.57, 1.57)	0.82
DMs	331	1.17 (0.74, 1.85)	0.5	1.24 (0.57, 2.71)	0.58
^***a***^Adjusted for child’s sex and exact age, maternal smoking during pregnancy, infant (0–12 months) being around smokers, season of birth (mold/wet/pollen/dry), mean daily PM_2.5_ during first 3 months of life, breastfeeding duration, signs of moderate/extensive mold at home visit (6 or 12 months), distance (≤ 150 m) from highway (6 or 12 months), and signs of cockroaches at home visit (6 or 12 months). ^***b***^Adjusted odds ratios reflect change per 10-fold increase in metabolite concentrations (the metabolites were modeled as log_10_-transformed variables). ^***c***^Urinary measurements were adjusted for specific gravity. After excluding mothers with missing covariate data, specific gravity–adjusted DAP concentrations were available for 241 mothers during the first half of pregnancy and for 313 mothers during the second half of pregnancy. ^***d***^Pregnancy average is derived from the average of the two measurements taken during pregnancy; for mothers who did not have both measurements, the average reflects the single measurement available. ^***e***^Any report on respiratory symptoms including exercise-induced coughing was also included under the respiratory symptoms variable; i.e., all children classified as positive for exercise-induced coughing were also classified as having respiratory symptoms.					

The concentrations of total DAPs, DEs, and DMs measured in child urine collected between the ages of 6 months and 5 years (AUC) were significantly associated with both reported respiratory symptoms and exercise-induced coughing at 5 and 7 years of age (total DAPs, aOR for a 10-fold increase in concentration = 2.53; 95% CI: 1.32, 4.86, *p* = 0.005 for symptoms; aOR = 5.40; 95% CI: 2.10, 13.91, *p* < 0.001, for coughing) (Table 4). Similar results were obtained when the AUC-based estimate of childhood exposure was derived for metabolite concentrations through 3.5 years of age only (see Supplemental Material, Table S2).

**Table 4:** Repeated measures of respiratory outcomes at ages 5 and 7 associated with the AUC of urinary DAP metabolites (nmol/g-creatinine) measured between 0.5 and 5 years of age [aOR (95% CI)].^*a,b*^

Childhood AUC	*n*	Respiratory symptoms	*p*-Value	Exercise-induced coughing^*c*^	*p*-Value
Total DAPs	270	2.53 (1.32, 4.86)	0.005	5.40 (2.10, 13.91)	< 0.001
DEs	270	2.35 (1.27, 4.34)	0.006	3.62 (1.38, 9.55)	0.009
DMs	270	2.17 (1.19, 3.98)	0.01	4.46 (1.81, 10.98)	0.001
^***a***^Adjusted for child’s sex and exact age, maternal smoking during pregnancy, infant (0–12 months) being around smokers, season of birth (mold/wet/pollen/dry), mean daily PM_2.5_ during first 3 months of life, breastfeeding duration, signs of moderate/extensive mold at home visit (6 or 12 months), distance (≤ 150 m) from highway (6 or 12 months), and signs of cockroaches at home visit (6 or 12 months). ^***b***^Adjusted odds ratios reflect change per 10-fold increase in metabolite concentrations (nmol/year/g creatinine) between 0.5 and 5 years of age as assessed by the AUC to summarize DAP concentrations over time during childhood (the metabolites were modeled as log_10_-transformed variables). For the AUC analysis (*n *= 270), we excluded children who did not have an AUC calculation (due to missing DAP measurements) and children with missing covariate data. ^***c***^Any report on respiratory symptoms including exercise-induced coughing was also included under the respiratory symptoms variable; i.e., all children classified as positive for exercise-induced coughing were also classified as having respiratory symptoms.					

Similar results were obtained when we did not adjust maternal DAP concentrations for specific gravity or child AUC levels for creatinine and when the models included both prenatal and child AUC measures in the same model (data not shown).

## Discussion

To our knowledge, the present study is the first prospective investigation of the relationship of prenatal and postnatal OP exposure and respiratory symptoms in children and the first study to investigate this relationship using a biomarker of exposure. Our results are consistent with findings from cross-sectional studies of associations between maternal report of early-life exposure to pesticides and asthma (Salam et al. 2004; Salameh et al. 2003). Our finding that prenatal exposure to OP pesticides as assessed by DAP metabolites in the second half of pregnancy, and particularly those that devolve to DE metabolites (e.g., chlorpyrifos, diazinon), is associated with increased odds of reported respiratory symptoms 5 to 7 years later is biologically plausible. OP pesticides can readily pass through the placenta (Rauh et al. 2006; Whyatt et al. 2009), and DAP metabolites can be found in amniotic fluid (Bradman et al. 2003). Furthermore, during the second half of pregnancy the alveoli are forming and surfactant is being synthesized; lung surfactant dysfunction is known to be related to the pathophysiology of asthma (Hameed et al. 2013; Wright et al. 2000).

We also found that postnatal exposure to OPs over the course of childhood was associated with higher odds of reported respiratory symptoms assessed at 5 and 7 years of age. These findings are consistent with previous results from our cohort, which showed that maternal work in agriculture during the child’s first year of life was associated with increased levels at age 2 years of Th2 cytokines, which are thought to play an important role in the development of asthma (Duramad et al. 2006).

Use of DAP metabolites as a marker of OP exposure is both a strength and a limitation of our study. Assessing exposure to specific individual OP pesticides is challenging because there are sensitive and specific assays for only a few parent compounds in blood or pesticide-specific metabolites in urine (e.g., TCPy for chlorpyrifos). DAPs are nonspecific metabolites commonly used as biomarkers in epidemiological studies; they represent an integrated measure of exposure to many OPs, reflecting the usual scenario in agricultural communities (Chen et al. 2012; Sudakin and Stone 2011). However, because OP pesticides can break down into DAPs in the environment, urinary DAP concentrations may reflect exposure both to the parent pesticide compounds and to preformed DAPs in food or dust (Lu et al. 2005).

In addition, because exposure to OP pesticides is highly variable, DAP metabolite levels may fluctuate considerably from day to day. These sources for exposure misclassification are nondifferential, and we assume that exposure misclassification resulting in bias toward the null may have potentially occurred in this study. Bias toward the null has been also previously suggested for associations between DAP measurements and health effects in children (Bradman et al. 2013). However, we assessed exposure to OP pesticides by measuring DAPs in urines collected twice during pregnancy and five times throughout early childhood, providing a better estimate of early-life exposure than single measurements.

This study has other strengths, particularly its longitudinal design and relatively large sample size. Furthermore, we used the well-established validated ISAAC questionnaire to interview the mothers about their children’s respiratory symptoms, and we adjusted for many covariates including exposure to other environmental agents and socioeconomic factors in the first year of life. Additionally, our study population was relatively homogeneous with regard to cultural and socioeconomic background, reducing the potential for uncontrolled confounding.

Our study was conducted within an agricultural community, and, as expected, the prenatal concentrations of urinary DAP metabolites in women from our study were higher than those in a representative U.S. sample of women of reproductive age (i.e., NHANES) (Bradman et al. 2005). In the current CHAMACOS study sample, the median of total maternal DAP concentrations among pregnant women was 127.5 nmol/L. NHANES median DAP levels, also measured between 1999 and 2000, were 72 nmol/L among pregnant women and 90 nmol/L among nonpregnant women of childbearing age (Bradman et al. 2005). Still, more than a quarter of the NHANES sample had DAP levels above the median levels measured in our current study, suggesting that the findings of this study have relevance for nonagricultural populations.

## Conclusions

Prevention and control of pediatric chronic respiratory diseases is a global health priority (Samoliński et al. 2012; WHO 2007), and it has been suggested that prevention should begin before childbirth (Samoliński et al. 2012). Although indoor use of most OPs was phased out by the U.S. EPA during the early to mid-2000s, these pesticides are still widely used in agriculture (Grube et al. 2011; U.S. EPA 2013). Our findings suggest that early-life exposure to OP pesticides is associated with respiratory symptoms consistent with a possible diagnosis of asthma among a population of children of primarily Mexican origin and living in an agricultural community in California. More research is needed to determine whether our findings are generalizable to other study populations as well as to further assess the possibility of susceptible period(s) and the mechanisms by which OP exposure may affect respiratory system development. Future studies on potential early-life exposure to pesticides should consider more objective measures of respiratory health such as spirometry.

## Supplemental Material

(228 KB) PDF
