# Supplementary material for: Early-life Exposure to Organophosphate Pesticides and Pediatric Respiratory Symptoms in the CHAMACOS Cohort
Source: Environ Health Perspect. 2014 Nov 4;123(2):179–85. doi: 10.1289/ehp.1408235 (PMC4314248; doi:10.1289/ehp.1408235)
Supplement: (228 KB) PDF [file ehp.1408235.s001.508.pdf]

Environmental Health Perspectives doi:10.1289/ehp.1408235

Supplemental Material

Early-life Exposure to Organophosphate Pesticides and Pediatric Respiratory Symptoms in the CHAMACOS Cohort

Rachel Raanan, Kim G. Harley, John R. Balmes, Asa Bradman, Michael Lipsett, and Brenda Eskenazi

Table S1. Percentage of maternal report[a] of the child's respiratory symptoms and use of asthma controller or rescue medications at 5 and 7 years of age, CHAMACOS cohort, California.

Respiratory symptoms or use of asthma medications	5 years	7 years
Wheezing or whistling in the chest	11.6 (40/344)	6.9 (24/347)
Wheezing, whistling, or shortness of breath so severe that the child could not finish saying a sentence	1.8 (6/343)	0.9 (3/347)
Trouble going to sleep or being awakened from sleep because of wheezing, whistling, shortness of breath	4.9 (17/344)	1.7 (6/347)
Trouble going to sleep or being awakened from sleep because of coughing that was not associated with a cold	16.0 (55/344)	9.5 (33/347)
Having to stop running or playing active games because of wheezing, whistling, shortness of breath	3.5 (12/343)	3.5 (12/347)
Having to stop running or playing active games because of coughing that was not associated with a cold (i.e., 'exercise-induced coughing')[b]	11.1 (38/343)	3.8 (13/347)
Use of asthma controller or rescue medications	9.0 (31/344)	6.3 (22/347)
Any report on respiratory symptoms or use of asthma controller or rescue medications (i.e., 'respiratory symptoms')	25.9 (89/344)	16.1 (56/347)

[a]Maternal report on child respiratory symptoms during the previous 12 months and on use of controller or rescue medication during the previous 12 months. [b]Any report on respiratory symptoms including exercise-induced coughing was also included under the respiratory symptoms variable, i.e., all children classified as positive for 'exercise-induced coughing' were also classified as having respiratory symptoms.

Table S2. Repeated measures of respiratory outcomes at ages 5 and 7 associated with the AUC of urinary DAP metabolites (nmol/g creatinine) measured between ages 0.5-3.5 years (excluding DAP metabolites measured at age five) [OR (95% CI)][a,b].

Childhood AUC	n	Respiratory symptoms aOR (95% CI)	p-value	Exercise-induced coughing[c] aOR (95% CI)	p-value
Total DAPs	235	2.24 (1.20, 4.16)	0.01	3.07 (1.23, 7.63)	0.02
DEs	235	2.54 (1.28, 5.03)	0.008	4.18 (1.54, 11.38)	0.005
DMs	235	1.81 (1.07, 3.05)	0.03	2.41 (1.07, 5.41)	0.03

Abbreviations: AUC (Area Under the Curve); Adjusted Odds Ratios (aOR); Confidence Intervals (CI); total dialkyl phosphate (DAP), diethyl (DE), and dimethyl (DM).

[a]Adjusted for child's sex and exact age, maternal smoking during pregnancy, infant (0-12 months) being around smokers, season of birth (mold/wet/pollen/dry), mean daily $PM_{2.5}$ during first 3 months of life, breast feeding duration, signs of moderate/extensive mold at home visit (6 or 12 months), distance (≤150m) from highway (6 or 12 months), and signs of cockroaches at home visit (6 or 12 months). [b]Odds ratios reflect change per 10-fold increase in metabolite concentrations (nmol/year/g creatinine) collected between 0.5 and 3.5 years of age (the DAP concentrations measured at age five were excluded from the AUC analysis) as assessed by the AUC to summarize DAP concentrations over time during childhood (the metabolites were modeled as log10- transformed variables). [c]Any report on respiratory symptoms including exercise-induced coughing was also included under the respiratory symptoms variable, i.e., all children classified as positive for 'exercise-induced coughing' were also classified as having respiratory symptoms.
